# March1 E3 Ubiquitin Ligase Modulates Features of Allergic Asthma in an Ovalbumin-Induced Mouse Model of Lung Inflammation

**DOI:** 10.1155/2018/3823910

**Published:** 2018-05-03

**Authors:** Osama A. Kishta, Antoine Sabourin, Leora Simon, Toby McGovern, Maxime Raymond, Tristan Galbas, Abdelilah Majdoubi, Satoshi Ishido, James G. Martin, Jacques Thibodeau

**Affiliations:** ^1^Université de Montréal, Laboratoire d'immunologie moléculaire, Département de microbiologie, Infectiologie et Immunologie, Montreal, QC, Canada H3T 1J4; ^2^McGill University Health Center, Meakins-Christie Laboratories for Lung Research, Montreal, QC, Canada 4HA 3J1; ^3^Department of Microbiology, Hyogo College of Medicine, 1-1 Mukogawa-cho, Nishinomiya 663-8501, Japan

## Abstract

Membrane-associated RING-CH-1 (March1) is a member of the March family of E3 ubiquitin ligases. March1 downregulates cell surface expression of MHC II and CD86 by targeting them to lysosomal degradation. Given the key roles of MHC class II and CD86 in T cell activation and to get further insights into the development of allergic inflammation, we asked whether March1 deficiency exacerbates or attenuates features of allergic asthma in mice. Herein, we used an acute model of allergy to compare the asthmatic phenotype of March1-deficient and -sufficient mice immunized with ovalbumin (OVA) and later challenged by intranasal instillation of OVA in the lungs. We found that eosinophilic inflammation in airways and lung tissue was similar between WT and March1^−/−^ allergic mice, whereas neutrophilic inflammation was significant only in March1^−/−^ mice. Airway hyperresponsiveness as well as levels of IFN-*γ*, IL-13, IL-6, and IL-10 was lower in the lungs of asthmatic March1^−/−^ mice compared to WT, whereas lung levels of TNF-*α*, IL-4, and IL-5 were not significantly different. Interestingly, in the serum, levels of total and ova-specific IgE were reduced in March1-deficient mice as compared to WT mice. Taken together, our results demonstrate a role of March1 E3 ubiquitin ligase in modulating allergic responses.

## 1. Introduction

Allergic asthma is a complex inflammatory disease, characterized by a Th2-skewed immune response [1]. Upon exposure of asthmatics to an allergen, antigen-presenting cells (APCs), such as dendritic cells (DCs) and macrophages, present peptides derived from the allergen to naïve CD4 T lymphocytes in the context of MHC class II molecules (MHC II), followed by costimulatory signals delivered by CD86. Both MHC II and CD86 are targeted for ubiquitination by March1, a member of the membrane-associated RING-CH (March) family of E3 ubiquitin ligases [2, 3]. Ubiquitinated MHC II and CD86 are targeted for lysosomal degradation, thereby inhibiting these molecules from recycling on cell surface in the resting state [2, 4]. Upon activation, March1 expression in DCs and B lymphocytes is inhibited to increase the stability of MHC II on the cell surface and maximize antigen presentation to naïve T cells [5–7]. In line with these observations, IL-10, a potent anti-inflammatory cytokine, stimulates March1 expression and consequently downregulates expression of MHC II in human primary monocytes and mouse bone marrow-derived macrophages [8–10]. Further, March1-mediated MHC II ubiquitination is required for DCs to produce antigen-specific regulatory T cells [11], which in turn impair DC function ability to activate CD4 T cells in an IL-10/March1-dependent manner [12]. These studies suggest that March1 may attenuate allergic reactions in vivo.

Paradoxically, bone marrow-derived conventional DCs (cDCs) from March1-deficient mice presented OVA peptide to naïve CD4 T cells in vitro efficiently, but their ability to activate CD4 T cells was significantly reduced compared to cDCs from March1-sufficient mice. This suppression was attributable to loss of MHC II (and not CD86) ubiquitination [7]. Moreover, Th1/Th17 differentiation of naïve CD4 T cells was inhibited when they were cocultured with March1-deficient cDCs [7]. According to these studies and considering the impact of MHCII/costimulators signals strength in T cell polarization [13], March1 deficiency may lead to impaired immune responses and modulate subsequent asthmatic features of allergy. Whether March1 deficiency attenuates, exacerbates, or modulates allergic lung inflammation in an in vivo model remains elusive. Thus, we assessed the responses of March1-deficient mice to sensitization and challenge with an allergen. More specifically, we addressed whether allergic lung inflammation, airway hyperresponsiveness, downstream cytokine profile, and mucus production were affected by March1 deficiency in vivo in a murine model of allergic asthma. Our results demonstrate that March1 deficiency leads to lung neutrophilic inflammation, in parallel with eosinophilia. It also reduces airway hyperresponsiveness as well as IL-13, IL-10, and IL-6 production, while it has no effect on OVA-induced eosinophilic lung inflammation, and mucus production.

## 2. Material and Methods

### 2.1. Mice

Colonies of wild-type C57BL/6J and March1-deficient mice (on a C57BL/6 background) [4] were maintained in our facility. All procedures were approved by the Université de Montréal Animal Use Committee according to the Canadian guidelines for animal care and use.

### 2.2. Ovalbumin (OVA) Model of Allergy

Allergic asthma was induced as described previously [14] with slight modifications in route and amount of allergen [15]. Briefly, female mice (6–10 weeks) were sham sensitized by intraperitoneal injection with 150 *μ*l of sterile PBS or sensitized with 40 *μ*g OVA (purity ≥ 98%, Sigma-Aldrich cat. number A5503-1G) adsorbed to 2 mg Imject™ Alum adjuvant (Thermo Fisher Scientific) in 150 *μ*l PBS on day 0 and 7. Then, all mice were challenged with OVA (100 *μ*g in 40 *μ*l PBS) intranasally under isoflurane anesthesia on days 14, 15, and 16. Mice were studied 24 h after the last challenge. Four experimental groups were studied: WT(sal-ova), WT(ova-ova), March1^**−/−**^(sal-ova), March1^−/−^(ova-ova).

### 2.3. Lung Cell Harvest and Flow Cytometry

On day 17, mice were euthanized with CO_2_. Lungs were removed and the right lobe was digested with collagenase D (Roche Diagnostics), as described before [16]. For intracellular staining, the whole lungs were digested with collagenase to ensure that sufficient numbers of cells were collected. Briefly, lungs were chopped into small pieces with scissors, immersed in 5 mL of collagenase D (200 unit/mL), Ca^2+^ and Mg^2+^, DNAase, and incubated at 37°C with shaking for 60 min. Cells were passed through 70 *μ*m pore mesh and washed once with PBS. Red blood cells were lysed with ammonium chloride. For surface marker staining, equal numbers of cells were incubated with survival stain (Zombie AquaTM, Biolegend) for 15 min. Cells were incubated with 20 *μ*l of rat serum on ice for 15 min to prevent nonspecific binding. Cells were then stained with various fluorochrome-conjugated antibody cocktails for 35 min and analyzed by flow cytometry on a FacsCanto II (BD Biosciences, CA). For intracellular staining of cytokines, 1.5–2 × 10^6^ cells per mouse were incubated at 37°C with cell activation cocktail (brefeldin A, PMA, and ionomycin; Biolegend) for 3.5 h. Cells were then washed and stained for survival with Zombie Aqua stain, as described above. Cells were surface stained for CD45 and CD4, washed, fixed with 4% formalin for 20 min on ice in the dark, washed once with PBS 0.1% BSA, and kept overnight at 4°C. Cells were then permeabilized with 0.2% saponin, stained with anti-IL-5 (PE), anti-IL-13 (PE-Cy7), and anti-IFN*γ* (APC) for 40 min, washed twice with in PBS 0.2% saponin, and analyzed by flow cytometry.

### 2.4. Antibodies

Antibodies from BD Biosciences: PE-anti-Siglec F (E50-2440), PE-Cy7-anti-CD19 (6D5), Percp 5,5-anti-CD11b (M1701), PE-anti-IL-5 (TRFK5), and APC-anti-IFN-*γ* (XMG1.2).

Antibodies from Biolegends: APC-anti-Ly6G (1A8), PE-CY7-anti-CD11c (HL3), BV421-anti-CD45 (30-F11), PE-anti-CD88, CR5a (20/70), and Alexa-fluor anti-I-A/I-E, MHC II (M5/114.15.2).

### 2.5. Lung Cytokine Measurement

Left lobes were frozen and homogenized in RIPA buffer containing protease inhibitors, spun at 10000 rpm for 5 min to remove debris, and supernatants were used to measure cytokines. The enhanced sensitivity cytometric bead array (CBA; BD Biosciences) was used following the manufacturer's instructions.

### 2.6. T Cell Stimulation Assay

Spleens were harvested aseptically, crushed in RPMI medium, and red blood cells were lysed in ammonium chloride. Cells were washed, resuspended in complete medium, and counted. Splenocytes were cultured in 96-well plate at 10^6^ cells/well in 200 *μ*L with OVA (1 mg/mL) or with medium alone for 3 days. Supernatants were then collected and frozen for later measurement of cytokines. Flex set CBA (BD Biosciences) was used following the manufacturer's instructions.

### 2.7. Dosage of Immunoglobulins

Total IgE and ova-specific IgE and IgG1 were measured in serum samples using commercial ELISA assays (eBiosciences), following the manufacturer's instructions.

### 2.8. Airway Hyperresponsiveness and Lung Lavage

On dedicated sets of mice, airway responsiveness to methacholine challenge was studied, as described previously [17]. Briefly, mice were anaesthetized and trachea was gently exposed. A blunt 18G needle was inserted in the trachea, secured and connected to flexiVent system. Methacholine provocation testing was performed. Respiratory system resistance was estimated using flexiVent software and the results were expressed as % of the baseline values. Mice were then killed by overdose of anesthesia and lungs were lavaged with 1 mL of PBS containing 0.1% (*w*/*v*) BSA.

### 2.9. Histology

On separate sets of mice, lungs were lavaged with 1 mL of PBS (0.1% BSA), then flushed with PBS, inflated with, and then immersed in 10% formalin for at least 24 h before embedding into paraffin blocks. Sections (4 *μ*m) were cut and Periodic Schiff Assay (PAS) staining of mucin was performed for evaluating mucus production [18, 19]. The slides were cover slipped and scanned using ScanScope®AT Turbo technology (Leica Biosystems Inc., CA USA) at 40x magnification. Lung sections were viewed digitally using Aperio ImageScope. Mucus positive cells were counted along the circumference of 2-3 airway sections. Circumference of these airway sections was measured using ImageJ software. Mucus cells were expressed as number of cells/*μ*m of airway circumference and the values of the captured sections from the same mouse were averaged.

Lung lavage samples, collected following airway hyperresponsiveness tests or before fixing lungs for histology, were spun at 2000 rpm for 10 min, resuspended in 0.5 mL PBS, counted on hemacytometer using trypan blue exclusion, and expressed as total cells/mouse BAL. Fifty *μ*l of each sample cells were adhered on slides using cytospin. Slides were then dried, fixed, and stained for differential count using HEMA 3 staining solutions (Fisher Scientific) as per the manufacturer's instructions. Cells (200–300) were counted under a light microscope (40x) based on the morphology of different leukocytes.

### 2.10. Statistical Analysis

Normally distributed data are presented as mean with standard error, whereas nonparametric data are presented as median with interquartile values. Difference between two groups in normally distributed data was analyzed by student's *t*-test, whereas the Mann–Whitney *U* test was used to analyze the difference between nonparametric data. A two-way ANOVA test was used to compare lung tissue inflammatory cells and airway resistance of all the experimental groups at each dose separately (fixed dose per test) considering strain and immunization factors. Airway resistance was also compared between ova-ova groups in the two strains (WT versus March1 KO) over the doses of methacholine challenge using two-way repeated measure ANOVA test, followed by Holm-Sidak tests for all pairwise multiple comparisons. A *p* value < 0.05 was considered statistically significant. ^∗^*p* < 0.05, ^∗∗^*p* < 0.01, and ^∗∗∗^*p* < 0.001. SigmaPlot V 11.0 was used to perform the statistical analysis and GraphPad Prism software was used for graphical plotting of data.

## 3. Results

In C57BL/6 mice, the OVA-induced acute model of allergy is characterized by Th2-driven pulmonary eosinophilic inflammation, elevated OVA-specific IgE and IgG1 as well as airway hyperresponsiveness [20]. However, some of these features and downstream levels of inflammatory mediators may vary depending on the dose of OVA used for sensitization as well as on the timing, dose, and method of challenge with the allergen [21]. Herein, we have compared these parameters between WT and March1^−/−^ mice sensitized by two i.p. injections of ova-alum complex followed by a challenge regimen consisting of 3 daily OVA nasal instillations starting on day 14, as described before [14, 15]. These mice are referred below as “ova-ova” and were compared to control mice, which received saline instead of OVA-Alum complex for the two i.p. injections. These sham-sensitized mice are referred to as “sal-ova” throughout the paper.

### 3.1. March1 Is Expressed in the Lung

**Figure 1:**
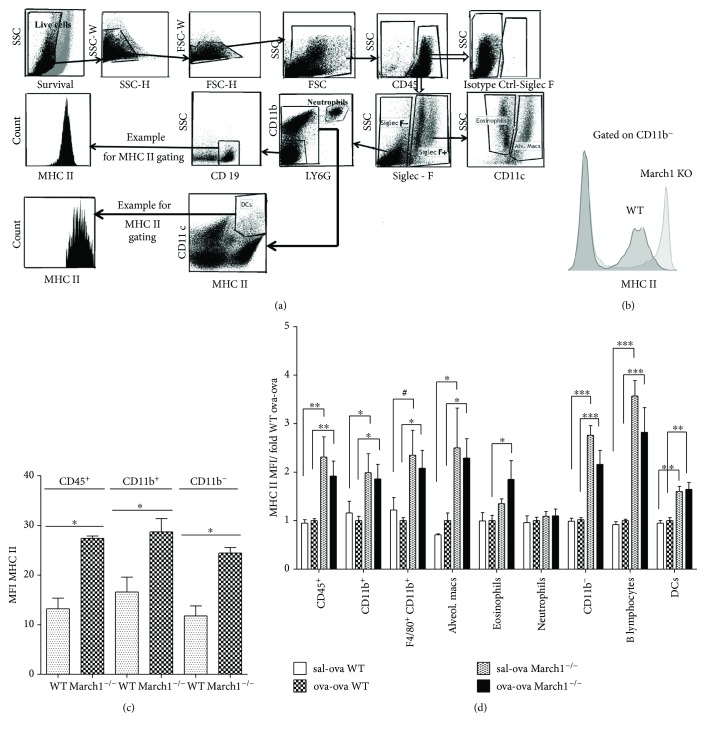
Deficiency of MARCH1 in MARCH1^−/−^ mice upregulates MHC II surface expression in lung myeloid and lymphoid immune cells. (a) Gating strategy illustrating analysis of the isolated and stained lung immune cells by flow cytometry for MHC II MFI assessment. (b) A representative comparison of MHC class II expression on WT and March1^−/−^ CD11b-negative cells is shown. (c) MHC class II MFI of lung cells from WT and March1^−/−^ mice. (d) MHC class II MFI of lung cells from WT and March1^−/−^ mice in OVA-induced allergic model. For each population, MFIs were normalized to the value obtained for WT mice sensitized and challenged with OVA. ^∗^*p* < 0.05, ^∗∗^*p* < 0.01, ^∗∗∗^*p* < 0.001, and ^#^*p* < 0.1 (trend). Data are presented as means with SEM; *n* = 4/group in (c) (one experiment); data are pooled from 4 independent experiments in (d), total *n* = 8-9/group.

According to the initial characterization of the March family members' tissue expression reported by Bartee et al. [22], March1 is mostly found in lymph nodes and spleen, followed by the lung. However, there are no reliable monoclonal antibodies capable of detecting this protein in primary cells and this last study relied on the mRNA abundance. As March1 mRNA is predominantly found in immature DCs, macrophages and B lymphocytes, we verified that it is indeed functional in lung hematopoietic cells using flow cytometry to monitor MHC class II expression. The gating strategy used to analyze single-cell lung suspensions from WT and March1^−/−^ mice is shown in Figure 1(a). First, we observed that MHC class II levels are higher in the bulk of CD45^+^ cells, independent of their expression of CD11b (Figures 1(b) and 1(c)). Then, we verified if MHC II expression could be modulated following exposure of immunized mice to ovalbumin in our model. MHC II surface expression was evaluated in lungs of sham-sensitized and challenged (sal-ova) as well as in sensitized and challenged (ova-ova) mice from both strains. Although MHC II surface expression was higher in CD45^+^, CD11b^+^, CD11b^−^, DCs, and B lymphocyte populations from lungs of March1^−/−^ as compared to WT mice, this increase was independent from immunization conditions (Figure 1(d)).

### 3.2. March1 Dampens Airway and Lung Neutrophilic Inflammation in Allergic Asthma without Affecting Eosinophilia

Airway and lung inflammation, characterized by the predominant accumulation of eosinophils is considered one of the hallmarks of allergic asthma in C57BL/6 [23]. We show that in airways, the OVA challenge of immunized mice induced a strong rise in total inflammatory cells, both in WT and March1^−/−^ mice (Table 1). However, this increase was more prominent in March1^−/−^ (*p* < 0.001) and tending to be higher in WT mice (*p* < 0.1). These cells were further characterized to identify various subpopulations based on their morphology. We found that eosinophils in airways of immunized and challenged groups (ova-ova) were significantly higher relative to their sham-immunized and challenged controls (sal-ova) in both strains, without significant difference between the March1-deficient and -sufficient mice. Neutrophils were higher in airways (BAL) of ova-ova relative to sal-ova within March1^**−/−**^ but not WT (Table 1). In both strains, lymphocytes were higher in ova-ova, relative to sal-ova (*p* < 0.1, Table 1). The more prominent neutrophilic cellular count in ova-ova March1^**−/−**^ mice suggests that neutrophilic inflammation contributes to the relatively higher BAL cellularity in the March1^−/−^ ova-ova group.

**Figure 2:**
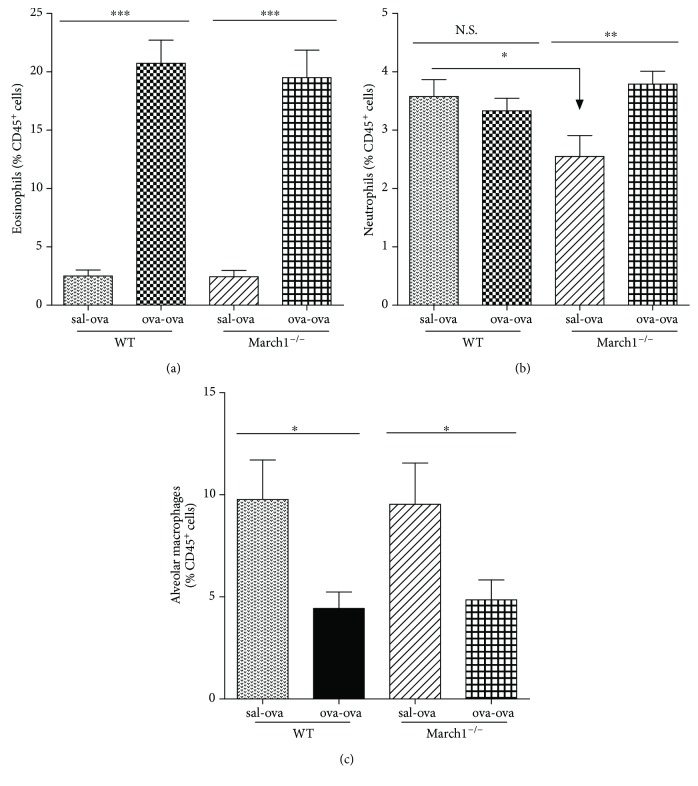
March1 does not affect allergic-induced eosinophilic recruitment or alveolar macrophages inflammatory dynamics in lung tissue. Lung immune cells were isolated by digestion and eosinophils (a), neutrophils (b), and alveolar macrophages (c) were analyzed by flow cytometry according to the gating strategy depicted in Figure 1(a). Cell numbers were normalized as percentage of CD45^+^ immune cells. ^∗^*p* < 0.05, ^∗∗^*p* < 0.01, ^∗∗∗^*p* < 0.001. Data are pooled from 5 independent experiments and presented as mean with SEM; *n* = 8–12/group.

To determine if the immune cell accumulation in BAL is accompanied by a similar increase in the tissue, lungs were analyzed by flow cytometry. Eosinophils (CD45^**+**^SiglecF^**+**^ CD11c^−^), neutrophils (CD45^**+**^Siglec F^−^ CD11b^**+**^ Ly6G^**+**^), and alveolar macrophages (CD45^+^ Siglec F^+^ CD11c^+^) were identified based on the gating strategy depicted in Figure 1(a) [24]. Interestingly, as seen in BAL, a strong accumulation of eosinophils was observed following the OVA challenge (ova-ova) but the two strains showed again no difference in the level of lung tissue eosinophilic inflammation (Figure 2(a)). Of note, a two-way ANOVA analysis of lung tissue neutrophils revealed a significant interaction between the two factors (strain and immunization), which was not the case for eosinophils, for example. All pairwise multiple comparison tests showed that neutrophil numbers in the lung tissue were significantly higher in ova-ova relative to sal-ova sham-sensitized controls within March1^**−/−**^, whereas in lungs of sal-ova and ova-ova (within WT) they are not significantly different. Likewise, within sal-ova groups, lung neutrophils were lower in March1^**−/−**^ than WT. Comparing neutrophils in sal-ova versus ova-ova within each strain separately indicates a consistent neutrophilia in both lung airways (BAL) and tissue of March1^**−/−**^ mice but not within WT groups (Table 1 and Figure 2(b)).

As alveolar macrophages are reported to be protective in some models of asthma [25], we verified if their numbers differed between WT and March1^**−/−**^ mice. Alveolar macrophages were identified by flow cytometry on sets of mice that did not undergo lung lavage.  Figure 2(c) reveals that alveolar macrophage proportions decreased significantly in ova-ova versus sal-ova conditions for both strains, in line with the establishment of a proinflammatory environment. However, we found no difference between the two strains. Altogether, these results demonstrate that the allergic-induced change in cellular components of eosinophils and alveolar macrophages in the lung is independent of March1 expression.

### 3.3. March1 Modulates Cytokine Production during Lung Allergic Inflammation

**Figure 3:**
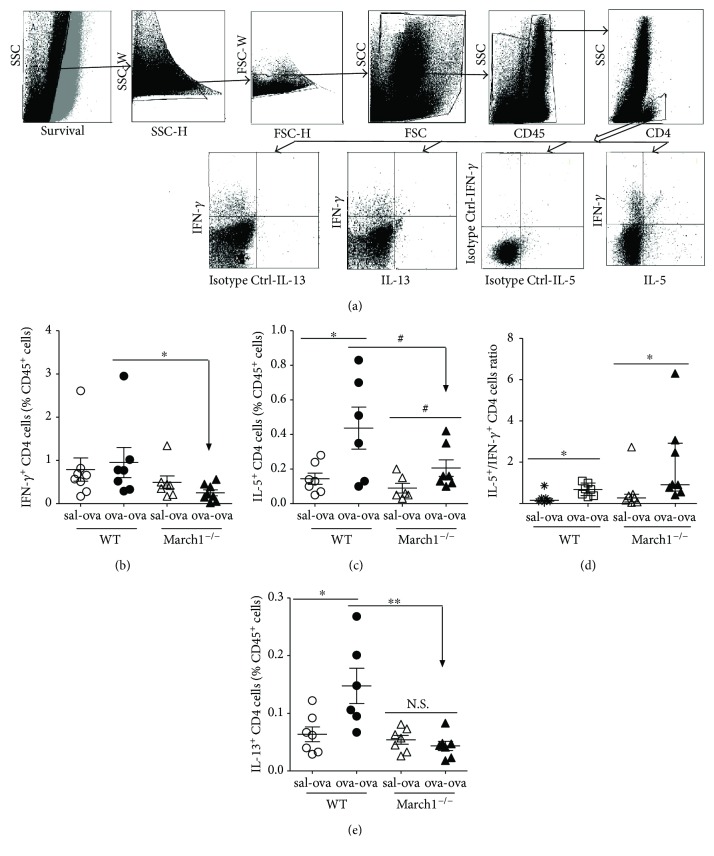
March1 deficiency is associated with less IFN-*γ*-, IL-5-, and IL-13-producing Th cells in the ovalbumin-allergic model. Lung immune cells were isolated by digestion and stained for surface CD45 and CD4 (a). Then, cells were permeabilized and stained for IFN-*γ* (b), IL-5 (c), and IL-13 (e). Gating strategy of lung cell subpopulations (a), IFN-*γ*-producing CD4 cells (b), IL-5-producing CD4 cells (c), ratio of IL-5/IFN-*γ* CD4 T cells (d), and IL-13-producing CD4 cells (e); all normalized and expressed as % of CD45^+^ immune cells. ^∗^*p* < 0.05, ^∗∗^*p* < 0.01, ^#^*p* < 0.1 (trend). Data are pooled from 3 independent experiments and presented in means with SEM (b, c) or medians with interquartile (d, e); *n* = 6–8/group.

In allergic patients and mice, the balance between Th2 CD4 T cells secreting IL-4, IL-5, IL-9, IL-10, and IL-13 and Th1 CD4 T cells secreting IFN-*γ* and IL-12 is shifted towards more Th2 CD4 cells [26]. The concentrations of Th2 cytokines in lung lavage and lung tissue homogenates are also reported to be higher than Th1 cytokines in allergic inflammation [1, 27, 28]. In order to determine if the OVA-induced inflammation described above was accompanied by a rise in Th2 cytokines, we first performed intracellular staining on lung cells to identify cytokine-producing T lymphocytes (Figure 3(a)). Interestingly, although the induction of allergic asthma did not result in an accumulation of the IFN-*γ*-producing Th1 cells in either strain, we found significantly less of those activated CD4 T cells (secreting IFN-*γ*) in ova-ova March1^**−/−**^ compared to ova-ova WT (Figure 3(b)). On the other hand, IL-5-expressing CD4 T cells were significantly higher in ova-ova compared to sal-ova controls in WT, whereas they were mildly elevated in March1 ^**−/−**^ ova-ova compared to their controls (sal-ova) (*p* < 0.1) (Figure 3(c)). However, the ratio of IL-5 over IFN-*γ*-expressing CD4^+^ cells in the lung was significantly higher in ova-ova compared to sham-sensitized and challenged (sal-ova) in both strains, without difference between WT and KO (Figure 3(d)).

**Figure 4:**
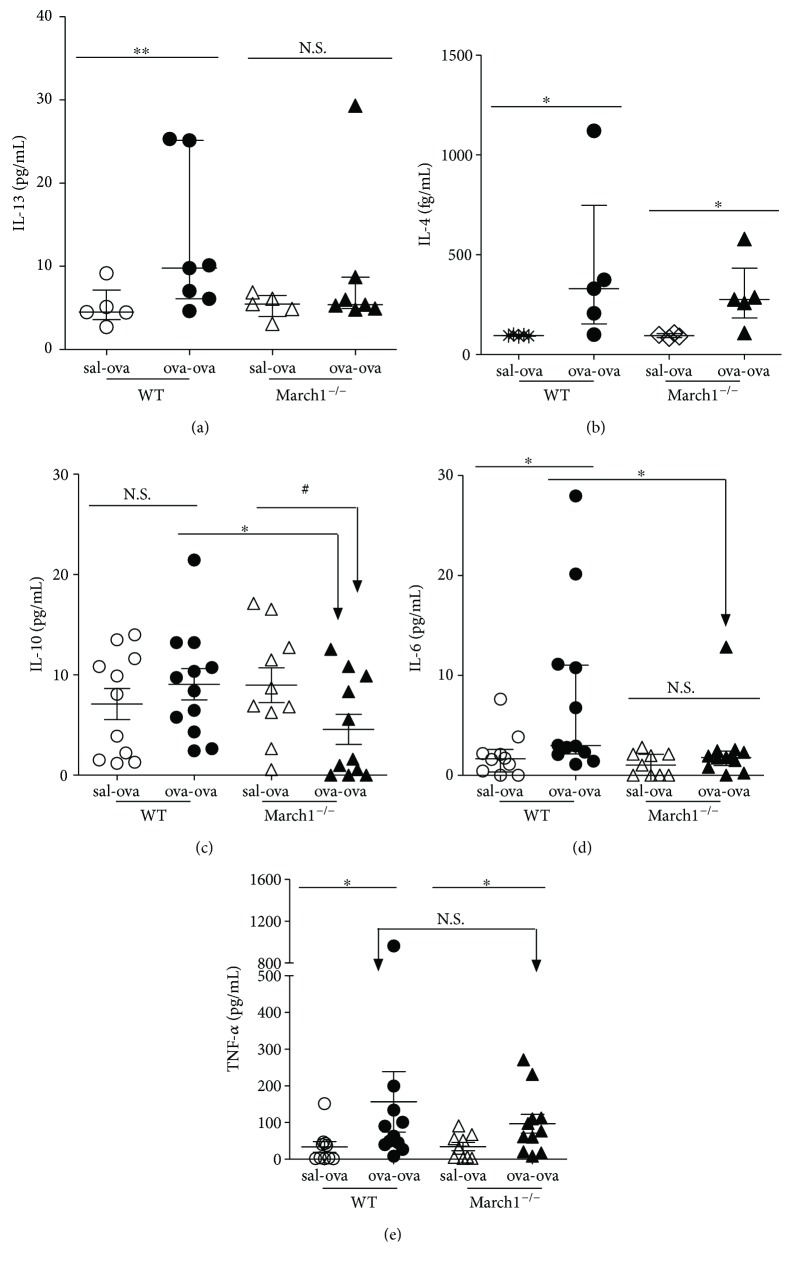
March1 deficiency is associated with less IL-13, IL-10, and IL-6 in lungs of OVA-sensitized and challenged mice. IL-13 (a), IL-4 (b), IL-10 (c), IL-6 (d), and TNF-*α* (e) cytokines were measured in lung homogenates using an enhanced sensitivity cytometric bead array (CBA). ^∗^*p* < 0.05, ^∗∗^*p* < 0.01, ^#^*p* < 0.1 (trend), N.S.: nonsignificant. Data are pooled from several independent experiments and presented in median with interquartile values (a, b, d) or in mean with SEM (c, e); *n* = 5–7 (a, b), *n* = 9–12 (c, d, e).

Another Th2 cytokine of interest is IL-13, which is responsible for airway remodeling, including mucus production [29], and contributes to the inflammatory response [30]. We found that IL-13-producing T cells were significantly higher in lungs of ova-ova group compared to the controls (sal-ova) in WT but not in March1^**−/−**^ groups (Figure 3(e)). Further, IL-13^+^ CD4 T cells in ova-ova WT group were also significantly higher than in March1^−/−^ mice counterparts (ova-ova) (Figure 3(e)). Interestingly, the pattern of IL-13 in lung homogenates was in conformity with that of the intracellular staining results of CD4^**+**^ producing IL-13, confirming the effect of March1 on this Th2 cytokine (Figures 3(e) and 4(a)). Altogether, these data demonstrate a Th2 bias in this model and suggest that March1 is required to maximize a Th2-skewed immune response and cytokine profile.

To extend our analysis, we measured protein levels of other cytokines in lung homogenates. In line with a Th2-skewed response in this allergic model, IL-4 in lung tissue homogenates was also significantly higher in ova-ova compared to sal-ova conditions for WT and March1^**−/−**^ mice. However, we could not observe a difference between the two strains (Figure 4(b)). The immunosuppressive cytokine IL-10 is produced by all subtypes of T cells, as well as B cells and myeloid cells [31]. Our results show that in lung homogenates, IL-10 concentrations tended to be diminished in ova-ova versus sal-ova in March1^**−/−**^ but not in WT mice (Figure 4(c)). Further, for the ova-ova groups, IL-10 was significantly less in March1^−/−^ than WT mice (Figure 4(c)).

We also tested the presence of two proinflammatory cytokines (IL-6 and TNF-*α*) in lungs. IL-6 is produced by a wide variety of cell types and is known to contribute to Th2 cytokine production, lung eosinophilia, and airway hyperresponsiveness [32]. As such, it was found to be higher in serum of asthmatic patients [33, 34] and mice [32]. In our study, as expected, IL-6 was significantly higher in lung homogenates from ova-ova WT group compared to the sal-ova controls. However, in March1^−/−^ mice, the IL-6 levels were low in both groups and were not affected by prior ova sensitization (Figure 4(d)). For TNF-*α*, which is known to contribute to several features of OVA-induced allergic airway inflammation [35], we observed an increase in ova-ova versus sal-ova controls both in WT and March1^**−/−**^ mice but without any significant difference between the two strains (Figure 4(e)). Altogether, these results suggest that March1 differentially impacts certain cytokines that are known to contribute to the establishment of allergic asthma in the OVA model.

### 3.4. March1 Regulates the Production of OVA-Specific Immunoglobulins (Igs)

**Figure 5:**
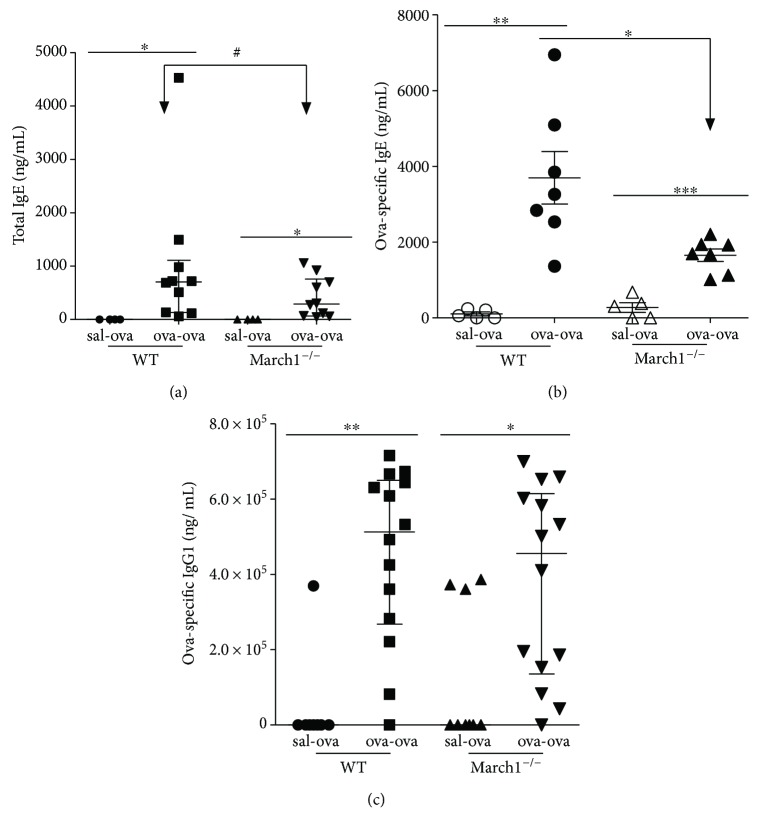
March1 deficiency leads to less antibody production against OVA in the ovalbumin-allergic model. Total IgE (a), OVA-specific IgE (b), and OVA-specific IgG_1_ (c) were measured in the serum of WT and March1^−/−^ mice. ^∗^*p* < 0.05, ^∗∗^*p* < 0.01, ^∗∗∗^*p* < 0.001, ^#^*p* < 0.1 (trend). Data are pooled from 4 independent experiments and presented as mean with SEM (a, b); *n* = 4–7. In (c) (IgG1), data are pooled from 7 independent experiments and presented as median with interquartile values; *n* = 8–14.

**Figure 6:**
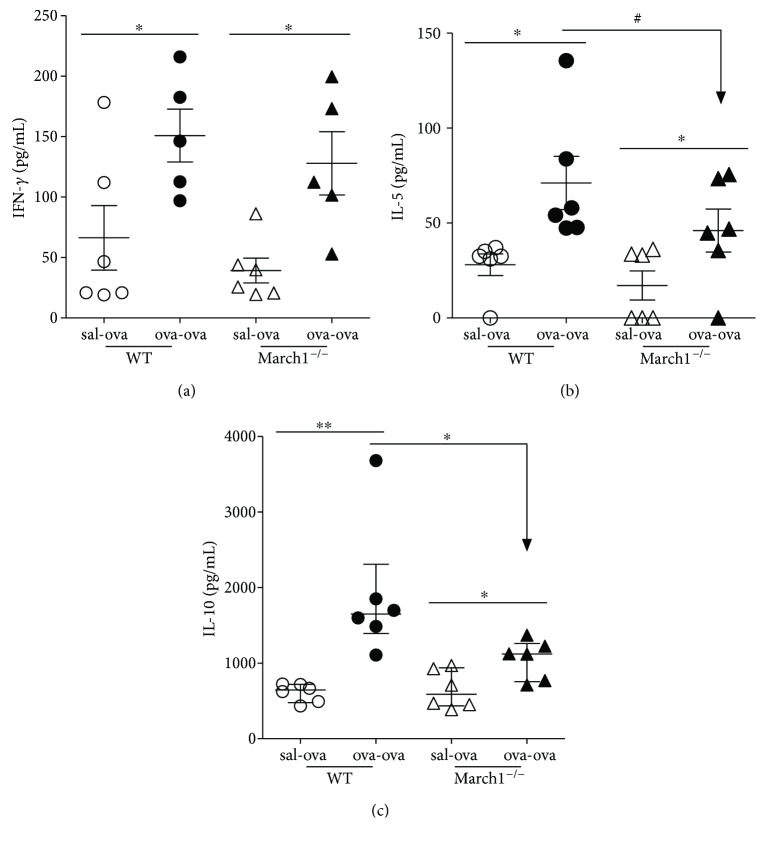
March1deficiency is associated with downregulation of IL-10 production. Splenocytes were isolated and cultured for 3 days with 1 mg/mL OVA. Supernatants were collected and assayed for IFN-*γ* (a), IL-5 (b), and IL-10 (c). ^∗^ and ^∗∗^ denote statistically significant. ^#^*p* < 0.1 (trend). Data are pooled from 3 independent experiments and presented as mean with SEM; *n* = 6–8/group.

An increase in total IgE as well as antigen-specific IgE and IgG_1_ antibodies is considered one of the hallmarks of the Th2 response leading to allergic asthma [36]. Total IgE and OVA-specific IgE and IgG_1_ were measured in serum. As compared to control mice, Ova sensitization and challenge resulted in a significant increase in seric total IgE concentrations in WT and March1^−/−^ mice (Figure 5(a)). However, the IgE concentrations tended to be less for ova-ova March1^−/−^ mice compared to their WT counterparts (Figure 5(a)). Interestingly, while both the Ova-specific IgEs and IgG1s were increased in the ova-ova groups (versus sal-ova within each strain), March1 was needed to obtain a maximal accumulation of OVA-specific IgEs (Figures 5(b) and 5(c)). These results suggest that that March1 somehow affects class-switching events and/or antibody generation. As antibody production is dependent on antigen presentation in secondary lymphoid organs, we tested the efficiency of OVA presentation in vitro using splenocytes from immunized animals. As judged by the IFN-*γ* production, our results showed an increased T cell activation in response to exogenously added OVA protein in all mice that have been sensitized and challenged in vivo (Figure 6(a)). Further, IL-5 level in supernatant of splenocyte cultures from ova-ova March1^**−/−**^ mice tended to be less than in WT ova-ova, in consistency with the lower level of IL-5^+^ CD4 cells in lungs of March1^**−/−**^ mice (Figure 6(b)). Interestingly, the OVA-induced IL-10 production was also reduced in splenocytes from March1-deficient mice, again, in line with the reduced IL-10 concentrations in lungs of March1^**−/−**^ ova-ova mice (Figure 6(c)). Altogether, these results suggest a role for March1 in the mucosal response to antigens and in maximizing Th2 cytokines.

### 3.5. March1 Modulates C5aR (CD88) Expression by Alveolar Macrophages and Lymphocytes

**Figure 7:**
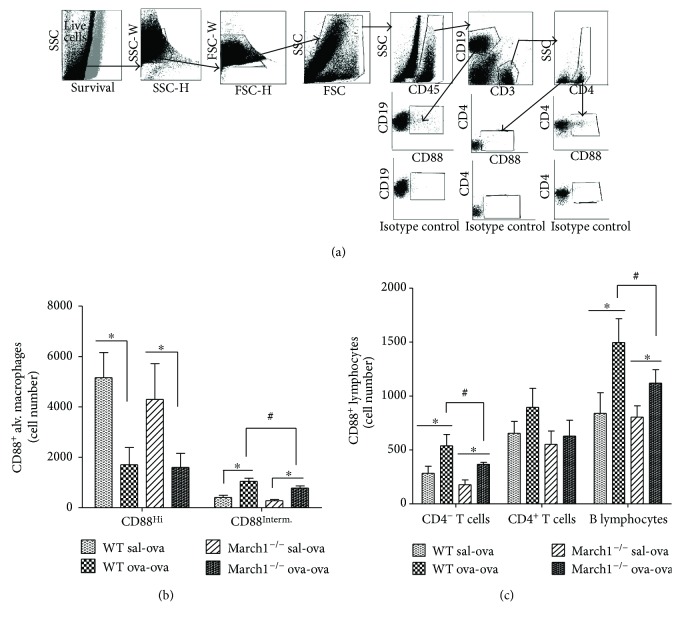
The OVA challenge of immunized mice is associated with an increase in the number of lung CD88^Interm.^ alveolar macrophages and CD88-positive lymphocytes relative to sham-sensitized groups in the two strains, the magnitude of which is reduced in the absence of March1. CD88-expressing lung lymphocytes were identified by flow cytometry (a). Alveolar macrophages were identified as in Figure 1(a). The numbers of CD88^Hi^ and CD88^Interm^ alveolar macrophages were expressed as absolute numbers (b). Absolute numbers of CD88-positive T cells and B lymphocytes are shown in (c). ^∗^ denotes statistically significant, ^#^*p* < 0.1 (trend). Data are pooled from 3 independent experiments and presented as mean with SEM; *n* = 5–8/group.

IgG_1_ antibodies play a key role in allergic asthma through activation of complement [36]. As March1 regulates the display of various transmembrane proteins at the plasma membrane, and given the established proinflammatory role of the complement by-product, C5a, in allergic asthma [36–38], we wondered if March1 could modulate the complement receptor (C5aR; CD88) expression in lung inflammatory cells as potential pathway in modulating allergic reactions. Therefore, we assessed CD88 expression by flow cytometry on lung inflammatory cells gated as in Figures 1(a) (myeloid cells) and 7(a) (lymphoid cells). OVA challenge of immunized mice resulted in switch of significant portion of CD88^+^ alveolar macrophages from CD88^Hi^ to CD88^int^ expressing cells in both strains; however, CD88^int^ alveolar macrophages, which significantly increased in ova-ova versus sal-ova within each strain, tended to be less in ova-ova March1^**−/−**^ relative to ova-ova WT (Figure 7(b)).

Since CD88 is known to be expressed mainly by myeloid cells, and to less extent by lymphocytes [39], it was not surprising in our study to find higher number of CD88^+^ alveolar macrophages (about two- to threefold more) compared to CD88^+^ lymphocytes. The proportions of CD88^+^ lymphocytes increased with OVA challenge of immunized mice in both strains, but only in B cells and CD4^−^ CD3^+^ cells this increase was significant; whereas CD88^+^ CD4^−^ T cells and B lymphocytes in March1^−/−^ ova-ova tended to be lower compared to ova-ova WT (Figure 7(c)), in consistency with the pattern of CD88^int^ alveolar macrophages (Figure 7(b)), suggesting an overall lower positive and expression of CD88 by both alveolar macrophages and lymphocytes in March1-deficent mice.

### 3.6. March1 Enhances OVA-Induced Airway Hyperresponsiveness without Affecting Mucus Production

**Figure 8:**
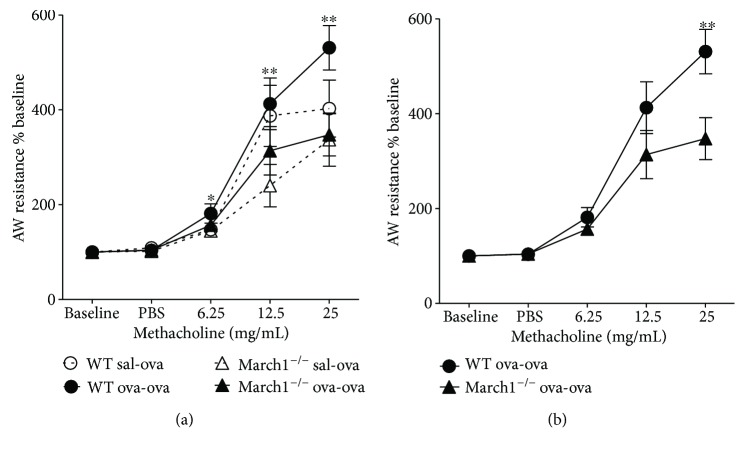
March1 enhances airway hyperreactivity in lungs of OVA-sensitized and challenged mice in the ovalbumin-allergic model. Mice underwent methacholine challenge by administration of gradual 3 doses of methacholine intranasally while they are under mechanical ventilation and airway resistance in WT and March1^−/−^ were recorded, extrapolated, and plotted (a). Airway resistance as % base for ova-ova groups in WT and March1^−/−^ was also plotted separately (b). Dotted line in (a) represents sal-ova and solid line represents ova-ova. ^∗^ and ^∗∗^ in (a) denote statistically significant for strain factor by 2-way ANOVA at each dose. In (b), ^∗∗^ denotes significant difference between ova-ova WT versus ova-ova KO at 25 mg/mL dose of methacholine. Data are pooled from 5 independent experiments and presented as means with SEM; *n* = 8-9/group. Further details on statistical analysis are laid out in the text.

Increased airway responsiveness to nonspecific stimuli, such as methacholine, is one of the hallmarks of an asthmatic reaction and can be measured in C57BL/6 mice [20, 23, 40]. In the present study, airway resistance was found to be significantly different among the experimental groups at each dose of methacholine challenge (two-way ANOVA test) (Figure 8(a)). By isolating and testing groups at each dose, airway resistance in March1^**−/−**^ (ova-ova) at 25 mg dose was significantly less than WT (ova-ova), *p* < 0.05, *t*-test. Further, comparing airway resistance between ova-ova groups in the two strains over doses of methacholine by two-way repeated measure ANOVA revealed that airway resistance tended to be different between the two strains (*p* = 0.059). All pairwise multiple comparison procedure confirmed that airway resistance is significantly lower in March1^**−/−**^ relative to WT at 25 mg/mL dose of methacholine challenge (*p* < 0.01) (Figure 8(b)).

**Figure 9:**
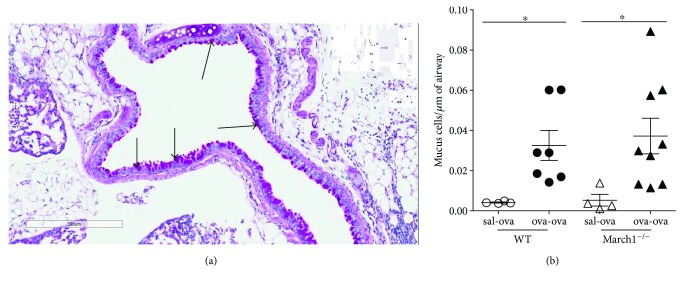
March1 does not affect mucus production in allergic model of lung inflammation. Lungs were fixed, embedded in paraffin, sectioned, and stained with PAS for mucus evaluation. (a) Representative image from WT (ova-ova) group. Pink cells along the circumference of airways were counted and expressed as number of cells/*μ*m of airway perimeter and plotted in panel (b). ^∗^ denotes statistically significant. Data are pooled from 3 independent experiments and presented as means with SEM; *n* = 4 (sal-ova), *n* = 7–9 (ova-ova).

Mucus production in the lung airways is another important hallmark of asthma [41, 42]. To determine if it is affected by March1 deficiency, we quantified mucus-secreting cells in lung tissues. Our results show that OVA sensitization greatly increased the number of mucus-producing cells in response to the OVA challenge but there was no difference in the magnitude of the response between WT and March1^−/−^ mice (Figures 9(a) and 9(b)).

### 3.7. Decreased Serum IgE and Lung IL-6 in March1^−/−^ ova-ova Is MHC II Ubiquitination Independent

**Figure 10:**
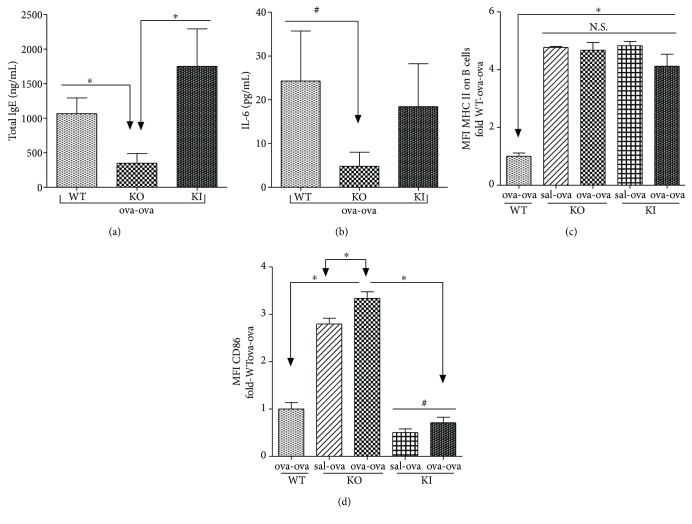
Decreased serum IgE, IL-13, and IL-6 in lungs of March1^−/−^ mice is not dependent on MHC II ubiquitination. Total serum IgE (a) and IL-6 in lung homogenates (b) were measured in ova-sensitized and challenged mice. Lung B cells were gated as in Figure 1(a) and the expression of MHC II (c) and CD86 (d) was assessed. ^∗^ denotes statistically significant, ^#^*p* < 0.1 (trend). Data are pooled from 2 independent experiments and presented as means with SEM; *n* = 4-5/group.

We asked whether the lower levels of IgE and IL-6 in March1^−/−^ are due to the upregulated surface expression of MHC II, caused by diminished ubiquitiniation by March1 or due to a different immune function. To address this question, we sensitized and challenged sets of WT and March1^−/−^ mice as well as MHC II knock-in (MHCII KI) mice (in which March1 is intact but the MHC II beta chain is mutated, preventing ubiquitination) [43]. We observed that IgE and IL-6 in ova-ova-treated MHCII KI mice were comparable to ova-ova WT and both WT and MHCII KI (ova-ova) are higher than March1^−/−^ (ova-ova) (Figures 10(a) and 10(b)). These MHCII KI mice, just like March1^−/−^, express more class II molecules at the surface of B cells (Figure 10(c)). However, they express CD86 to the same level as WT cells (Figure 10(d)). These results suggest that the increase in MHC II expression observed in March1^−/−^ mice is not sufficient to negatively regulate IgE and IL-6 production.

## 4. Discussion

IL-10, a potent regulatory cytokine, induces March1 expression in monocytes, macrophages, and DCs [9, 10, 44]. March1, through its ubiquitin ligase activity, displays immune-regulatory properties through downregulation of MHC II surface expression [9, 45]. The effect of MHC II ubiquitination on antigen presentation was studied by different groups using March1-deficient mice and MHCII knock-in mice (in which the sole MHCII *β* chain cytoplasmic lysine residue is mutated), resulting in conflicting findings (reviewed in [46]. Work by Ohmura-Hoshino et al. demonstrated that although loss of MHC II ubiquitination increased antigen presentation, it decreased the capacity of March1 KO and MHCII KI DCs to stimulate naïve T cell activation and differentiation in vitro due to downregulation of LFA-1 [7, 43]. However, McGehee et al. reported that MHCII ubiquitination in BM-DCs is not necessary for stimulation of OVA-specific T cells ex vivo [47]. Due to the broad expression of MHC II in multiple cell types during many cellular processes, it is important to evaluate the impact of March1 deficiency in the context of a Th2-mediated disease, such as allergic asthma.

Our results show that in all conditions, the expression of MHCII remains lower in WT APCs as compared to their March1^**−/−**^ counterparts_._ This demonstrates that March1 expression is not downmodulated by the ova sensitization and challenge in WT animals. Interestingly, such strong MHCII expression in March1-deficient mice did not exacerbate allergic asthma. While eosinophilic inflammation and mucus production were induced to a similar extent in March1^−/−^ and WT mice, neutrophilic inflammation was more prominent in March1^−/−^ relative to WT. Airway hyperresponsiveness, another hallmark of allergic asthma, was reduced in the absence of March1. Thus, one possibility to investigate in the future is that the increased expression of both MHCII and CD86 in March1-deficient mice blunts the Th_2_ response to the allergen and/or activates Th17 cells, which are known to increase neutrophil influx in airways of mice with allergic asthma [48]. Our results suggest a protective role of March1 from lung neutrophilic inflammation in allergic asthma.

IFN-*γ* is a Th1 cytokine, reported to suppress allergic reaction by a variety of mechanisms such as regulating allergen presentation to T lymphocytes, enhancing differentiation of naïve T lymphocytes into Th1, and inhibition of Th2 lymphocyte differentiation and suppression of Th2 cytokine release from Th2 activated cells [49]. Based on our results of less IFN-*γ* in March1^−/−^ and given that IFN-*γ* is a pleiotropic cytokine, it is apparent that the immune-suppressive function of INF-*γ* does not explain the lower level of this Th1 cytokine in March1-deficient mice. Since INF-*γ* regulates MHC II expression in different cell populations [50, 51], we speculate that its effector function in March1-deficient mice, which exhibited milder reaction in allergic and cytokine responses compared to WT, implicates regulation of MHC II expression by immune and nonimmune lung cells [52]. Future studies are needed to further explore the role of IFN-*γ* in this context.

Of note, March1-deficient T cells produced less IL-10, in line with the reduced levels of IL-10 detected in lungs of asthmatic March1^−/−^ mice. IL-10 has been reported to promote the development of allergen-induced smooth muscle hyperresponsiveness in C57BL/6 mice [53]. Interestingly, this could be due to the reduced IL-13 levels found in the lungs of March1 KO mice. Indeed, IL-10 production was shown to be reduced in Th2 cultures from IL-13-deficient mice [54]. Multiple mechanisms have been evoked to explain how IL-13 can increase and IFN-*γ* can downmodulate inflammation of the airways [49, 55]. IL-13 plays a critical role in allergies and exogenous IL-13 can induce AHR in absence of T and B cells [56, 57]. Further, it was previously found that AHR failed to develop in ovalbumin- sensitized and challenged mice deficient in IL-13, despite the robust Th2-biased pulmonary eosinophil response [56, 58]. Thus, the lower level of IL-10 and IL-13 may contribute to the mild airway hyperresponsiveness in March1^**−/−**^ compared to WT mice. The fact that this decrease in airway resistance in March1^**−/−**^ is evident (statistically significant) at a dose of 25 mg/kg methacholine can be attributed to one or both of the following reasons. First, C57BL/6 strain of mice is known to exhibit less AHR in an allergen model compared to other strains such as BALB/C [20], which poses limitation on studying this parameter and demands trying other strains and/or model modifications. To our knowledge, March1 deficiency has, so far, only been studied in the C57BL/6 background. Future studies should use BALB/C mice as a strain, more prone to AHR. Second, 25 mg/kg methacholine dose could be the optimum dose to reach detectable difference in airway resistance between WT and March1^−/−^ in C67BL/6 mice in this experimental setting.

An important issue for future studies will be to determine how the lack of March1 can modulate IL-13 production. As March1-deficient DCs were previously shown to produce aberrant cytokine profiles in response to TLR stimulation, the Th1/Th2 balance may be perturbed following OVA presentation. IL-13 is also produced by innate lymphoid cells (nuocytes, ILC2s), which express MHC class II and help shape T cell responses that control airway inflammation [59, 60]. Future studies should investigate their levels of March1 and how this ubiquitin ligase can potentially modulate the production of IL-13 or their capacity to trigger a Th2 type of response. Indeed, a March1-triggered reduced ligand density on ILC2s and professional APCs would limit TCR engagement and predispose for differentiation of Th2 cells [13, 61]. In support of this notion, a recent study using an in vivo model of allergy reported that limiting MHC II expression to DCs impairs the ability of the host to develop Th2 allergic immune response [62].

The CD88 complement receptor (C5aR) is a G-protein-coupled receptor, expressed mainly in myeloid cells and has been reported as a therapeutic target, which blockade reduced airway inflammatory cell and cytokine responses in OVA-murine model of allergic asthma [63]. It is stimulated by the complement by-product, C5a, which has multiple proinflammatory effector functions as reviewed before [36]. C5a, along with the other anaphylatoxin, C3a, is well known to be potent proinflammatory mediators contributing to allergic reactions through a variety of immune functions [64]. For example, C5a is chemoattractant for inflammatory cells (macrophages, neutrophils, basophils, and mast cells) [65]. Further, airway hyperreactivity, in IgG immune complex-induced acute lung injury, was reported to be C5 and C5a dependent [66]. Thus, lower CD88-expressing alveolar macrophages, CD4^−^ T cells, and B lymphocytes in ova-ova March1^**−/−**^ relative to WT in our study may further explain and contribute to the milder airway hyperresponsiveness in March1^**−/−**^ mice.

Another feature of asthma in mice [67] and humans, to be addressed and thought about in future studies, in relevance to IL-13 in March1 KO mice in this model is subepithelial lung fibrosis [41]. IL-13 is a potent activator of collagen production by fibroblasts in mice and human cells [56, 68]. Our results suggest that March1^−/−^ mice may exhibit less asthma-associated subepithelial lung fibrosis. In support of this notion, IL-6, a profibrotic cytokine in asthma model [41] was also lower in March1^**−/−**^ ova-ova. Whether this suggested protection from subepithelial lung fibrosis and from lung neutrophilic inflammation are related remains a valid question to be investigated for further characterization of March1's role in the immune disorder of allergic asthma.

March1 deficiency had a strong impact on the antibody response. We observed lower levels of total and ova-specific IgE and IgG_1_ in sera of March1^**−/−**^ mice relative to WT. As both IL-13 and IL-4 promotes IgE class switching [69], the lower IgE levels in March1^−/−^ could be attributed to less IL-13 in March1^−/−^ mice.

We observed that IgE and IL-6 in KI mice were comparable to ova-ova WT and both WT and KI (ova-ova) are higher than March1^**−/−**^ (ova-ova), suggesting that these effects are due to March1's activity on other targets (Figures 10(a) and 10(b)). This conclusion further confirms that other immune functions of March1 (beyond ubiquitination of MHC II) are more important in modulating the allergic reactions in this model.

In conclusion, we show here in this study that March1 E3 ubiquitin ligase contributes to the development of allergic inflammatory response by promoting Th2 cytokines, particularly those implicated in IgE class switching and airway hyperresponsiveness (IL-13). Our study also suggests that March1 promotes allergic reactions via enhancing the allergen-induced expression of complement receptor CD88 by inflammatory cells. This study also suggests that March1 can dually play a protective role from lung neutrophilic inflammation in allergic asthma. The conclusion of this study is expected to better direct future studies towards therapeutic targets for March1 in disease models and subsequent clinical perspectives.

## Figures and Tables

**Table 1:** March1 does not affect eosinophilic airway inflammation but induces neutrophilic inflammation in the airways: Total inflammatory cells in bronchoalveolar lavage (BAL) fluid were counted using hemocytometer and trypan blue exclusion method. Differential cell count in BAL: Differential cell count was performed on BAL cells adhered to slides and stained with HEMA-3. For each sample, 200–300 cells were counted under the microscope (40x), and their percentage was multiplied by the total cell count to extrapolate absolute differential count. ^∗^ and ^∗∗∗^ denote statistically significant; ^#^*p* < 0.1 (trend); N.S.: non-ignificant. Data are presented as median (nonparametric data) and pooled from 5 independent experiments. *N* = 9–12/group.

	WT median cells ×10^4^		*p* value WT sal-ova versus ova-ova	March1^−/−^ median cells ×10^4^		*p* value March1^−/−^ sal-ova versus ova-ova	*p* value ova-ova WT versus ova-ova March1^−/−^
	sal-ova	ova-ova		sal-ova	ova-ova		
Total cells/mouse BAL	6	15.9	0.09#	4	15.7	<0.001^∗∗∗^	N.S.
Mono/macs	5.16	4.9	N.S.	3.7	4.7	N.S.	N.S.
Lymphocytes	0	0.24	0.06#	0.01	0.28	0.06#	N.S.
Neutrophils	0	0.01	N.S.	0	0.27	0.02^∗^	N.S.
Eosinophils	0	1.9	<0.001^∗∗∗^	0.07	5.8	<0.001^∗∗∗^	N.S.

## Abbreviations

March1: Membrane-associated Ring CH 1
OVA: Ovalbumin
APCs: Antigen-presenting cells
DCs: Dendritic cells
BAL: Bronchoalveolar lavage
CBA: Cytometric bead array.
